# Supplementary material for: Correlates of the country differences in the infection and mortality rates during the first wave of the COVID-19 pandemic: evidence from Bayesian model averaging
Source: Sci Rep. 2022 May 2;12:7099. doi: 10.1038/s41598-022-10894-6 (PMC9058748; doi:10.1038/s41598-022-10894-6)
Supplement: Supplementary file 1 — Supplementary Information 1. [file 41598_2022_10894_MOESM1_ESM.pdf]

Supplementary Information for: Correlates of the country differences in the infection and mortality rates during the first wave of the COVID-19 pandemic: Evidence from Bayesian model averaging

Viktor Stojkoski[1,2,*], **Zoran Utkovski**[3,2], **Petar Jolakoski**[1], **Dragan Tevdovski**[1], **and Ljupcho Kocarev**[2,4]

[1]Faculty of Economics, Ss. Cyril and Methodius University in Skopje
[2]Macedonian Academy of Sciences and Arts
[3]Fraunhofer Heinrich Hertz Institute, Berlin
[4]Faculty of Computer Science and Engineering, Ss. Cyril and Methodius University in Skopje
[*]vstojkoski@eccf.ukim.edu.mk

ABSTRACT

The COVID-19 pandemic resulted in great discrepancies in both infection and mortality rates between countries. Besides the biological and epidemiological factors, a multitude of social and economic criteria also influenced the extent to which these discrepancies appeared. Consequently, there is an active debate regarding the critical socio-economic and health factors that correlate with the infection and mortality rates outcome of the pandemic. Here, we leverage Bayesian model averaging techniques and country level data to investigate whether 28 variables, which describe a diverse set of health and socio-economic characteristics, correlate with the final number of infections and deaths during the first wave of the coronavirus pandemic. We show that only a few variables are able to robustly correlate with these outcomes. To understand the relationship between the potential correlates in explaining the infection and death rates, we create a Jointness Space. Using this space, we conclude that the extent to which each variable is able to provide a credible explanation for the COVID-19 infections/mortality outcome varies between countries because of their heterogeneous features.

Contents

S1 Government response index . . . 1
S2 Data description . . . 2
Individual indices
S3 Bayesian model averaging . . . 5
S4 Robustness checks . . . 6
BMA outliers check • Alternate end date of the first wave • BMA quasi-Poisson specification • BMA Spatial autocorrelation check
S5 Construction of the coronavirus correlates Jointness space . . . 11
References **11**

S1 Government response index

To calculate our government response measure, we make use of Oxford's daily government response index. Oxford's daily government response index measures, on a scale of 1-100, the variation in daily government responses to COVID-19 by accumulating ordinal data on country social distancing measures on school, workplace and public transport closure; cancellation of public events; restrictions of internal movement; control of international travel and promotion of public campaigns on prevention of coronavirus spread; testing policies and procedures implemented for tracing contacts of infected individuals. We refer to[1] for a detailed overview on how the daily index is constructed.

To calculate the overall government response index $c_i(d_i^*)$ at the final date d_i^* from the provided daily indexes we implement the following procedure. Let $C_i(t)$ represent the government response on day t, where $t = 1, 2 \ldots d_i^*$, then our index can be

estimated as

$$c_i(d_i^*) = \sum_{s=1}^{d_i^*} w_i(s) C_i(s), \tag{S1}$$

where $w_i(s)$ are the weights given to each day since the first registered case. We use a simple inverse weight procedure by giving larger weights to earlier dates, i.e.,

$$w_i(s) = \frac{1}{s} / \sum_{k=1}^{d_i^*} \frac{1}{k}. \tag{S2}$$

We choose the last date d_i^* to be the last day at which the daily government response index $C_i(t)$ is at its maximum value.

S2 Data description

The data for the dependent variables are taken from Our World in Data coronavirus tracker. The tracker offers daily coverage of country coronavirus statistics, by collecting data mainly from the European Centre for Disease Prevention and Control. Because national aggregates often lag behind the regional and local health departments' data, an important part of the data collection process consists in utilizing thousands of daily reports released by local authorities. The results were made with data gathered on 13th November 2020.

The data used for measuring the possible health, social and economic correlates are gathered from 9 various sources. In particular, the collection is as follows: 19 variables are from the World Bank's World Development Indicators (WDI), 2 variables are from the Our World in Data database and there is 1 variable from World Bank's Environmental, social and governance data (ESG), the Worldometers database (WM), Data For Good (DFG), the State of Global Air (SGA), the Global footprint network (GFN), United Nations (UN) database and Google. Six of the potential correlates were constructed by deriving our own index with data taken from the described source. The construction procedures for each of these variables are described in the following subsection. The full list of sources together with links to their websites is given in Table S1. The data used in the analysis are available at `https://github.com/pero-jolak/coronavirus-socio-economic-determinants`.

Source	Link
COVID-19 infections/deaths	ourworldindata.org/coronavirus
DFG	dataforgood.fb.com
Google	maps.google.com
ESG	datacatalog.worldbank.org/dataset/environment-social-and-governance-data
GFN	data.footprintnetwork.org
Gov. Response	covidtracker.bsg.ox.ac.uk
Our world in data	ourworldindata.org
SGA	www.stateofglobalair.org/engage
UN	data.un.org
WDI	data.worldbank.org/
WGI	info.worldbank.org/governance/wgi
WM	/www.worldometers.info/world-population

Table S1. List of data sources.

To reduce the noise from the data we, use only data for countries with population above 1 million. In addition, we only use countries for which there is data on all of the potential correlates. Table S2 gives the countries for which all of these data was available.

Country	First Date	End Date	Country	First Date	End Date	Country	First Date	End Date
Albania	09-Mar	31-May	Georgia	27-Feb	26-Apr	Nigeria	28-Feb	03-May
Argentina	04-Mar	25-Apr	Ghana	13-Mar	17-Apr	Netherlands	28-Feb	10-May
Australia	25-Jan	27-Aug	Greece	27-Feb	13-Sep	Norway	27-Feb	19-Apr
Austria	26-Feb	13-Apr	Guatemala	15-Mar	26-Jul	Nepal	25-Jan	18-Aug
Azerbaijan	29-Feb	05-Aug	Honduras	12-Mar	07-Jun	New Zealand	28-Feb	27-Apr
Belgium	04-Feb	04-May	Croatia	26-Feb	26-Apr	Pakistan	27-Feb	03-Jun
Benin	17-Mar	10-May	Hungary	05-Mar	03-May	Panama	10-Mar	11-Oct
Burkina Faso	11-Mar	04-May	Indonesia	02-Mar	02-May	Peru	07-Mar	10-May
Bangladesh	09-Mar	30-May	India	30-Jan	19-Apr	Philippines	30-Jan	30-Apr
Bulgaria	08-Mar	30-Apr	Ireland	01-Mar	29-Oct	Papua New Guinea	21-Mar	11-Aug
Bosnia and Herzegovina	06-Mar	23-Apr	Iraq	25-Feb	26-Aug	Poland	04-Mar	01-Nov
Bolivia	12-Mar	23-Jun	Israel	22-Feb	16-Apr	Portugal	03-Mar	03-May
Brazil	26-Feb	28-Jul	Italy	31-Jan	03-May	Paraguay	08-Mar	24-May
Botswana	01-Apr	07-May	Jamaica	12-Mar	30-May	Romania	27-Feb	10-May
Canada	26-Jan	11-Aug	Jordan	03-Mar	20-Apr	Russia	01-Feb	31-May
Switzerland	26-Feb	29-May	Japan	15-Jan	13-May	Rwanda	15-Mar	03-May
Chile	04-Mar	04-Oct	Kazakhstan	15-Mar	10-May	Senegal	03-Mar	10-May
Côte d'Ivoire	12-Mar	07-May	Kenya	14-Mar	22-Jun	Singapore	24-Jan	01-Jun
Cameroon	07-Mar	30-Apr	Kyrgyzstan	19-Mar	29-Apr	El Salvador	19-Mar	01-Jun
Colombia	07-Mar	05-May	Korea	20-Jan	17-Apr	Serbia	07-Mar	20-Apr
Costa Rica	07-Mar	30-Apr	LAOS	25-Mar	03-May	Slovakia	07-Mar	29-Oct
Cyprus	10-Mar	03-May	Lithuania	28-Feb	13-Apr	Slovenia	05-Mar	19-Apr
Czechia	02-Mar	01-Apr	Latvia	03-Mar	11-May	Sweden	01-Feb	12-Jun
Germany	28-Jan	02-May	Morocco	03-Mar	10-Jun	Togo	07-Mar	07-Jun
Denmark	27-Feb	21-May	Moldova	08-Mar	15-May	Thailand	13-Jan	02-May
Dominican Republic	02-Mar	17-May	Madagascar	21-Mar	19-Apr	Trinidad and Tobago	13-Mar	30-Apr
Ecuador	01-Mar	03-May	Mexico	14-Jan	26-Oct	Turkey	12-Mar	20-Sep
Egypt	15-Feb	06-Jun	Myanmar	18-Mar	24-Oct	Tanzania	17-Mar	17-May
Spain	01-Feb	16-May	Mongolia	10-Mar	07-May	Uganda	22-Mar	17-May
Estonia	28-Feb	07-May	Mozambique	23-Mar	12-Jul	Ukraine	04-Mar	21-May
Ethiopia	14-Mar	10-Sep	Mauritius	20-Mar	14-May	USA	21-Jan	14-Jun
Finland	30-Jan	13-Apr	Malawi	03-Apr	31-Aug	Venezuela	15-Mar	01-Nov
France	25-Jan	25-May	Malaysia	25-Jan	09-Jun	Viet Nam	24-Jan	14-Apr
Gabon	13-Mar	15-Oct	Namibia	15-Mar	04-May	South Africa	06-Mar	31-May
UK	01-Feb	03-Nov	Niger	21-Mar	12-May	Zambia	19-Mar	07-May

Table S2. List of countries and estimation period.

Altogether, we end up with data on 28 variables and 105 countries. Table S3 reports the summary statistics of each variable. We hereby point out that as a measure of the correlate the log of the last observed value is taken (the value in 2019), unless otherwise stated in Table S3. This prevents the possible problem of endogenous independent variables in the specification of the regression.

In Fig. S1 we plot the correlation matrix between the potential correlates. It can be observed, that in general the correlation between the variables is large. Out of 378 variable pairs, 102 have correlation that is either below -0.6 or above 0.6.

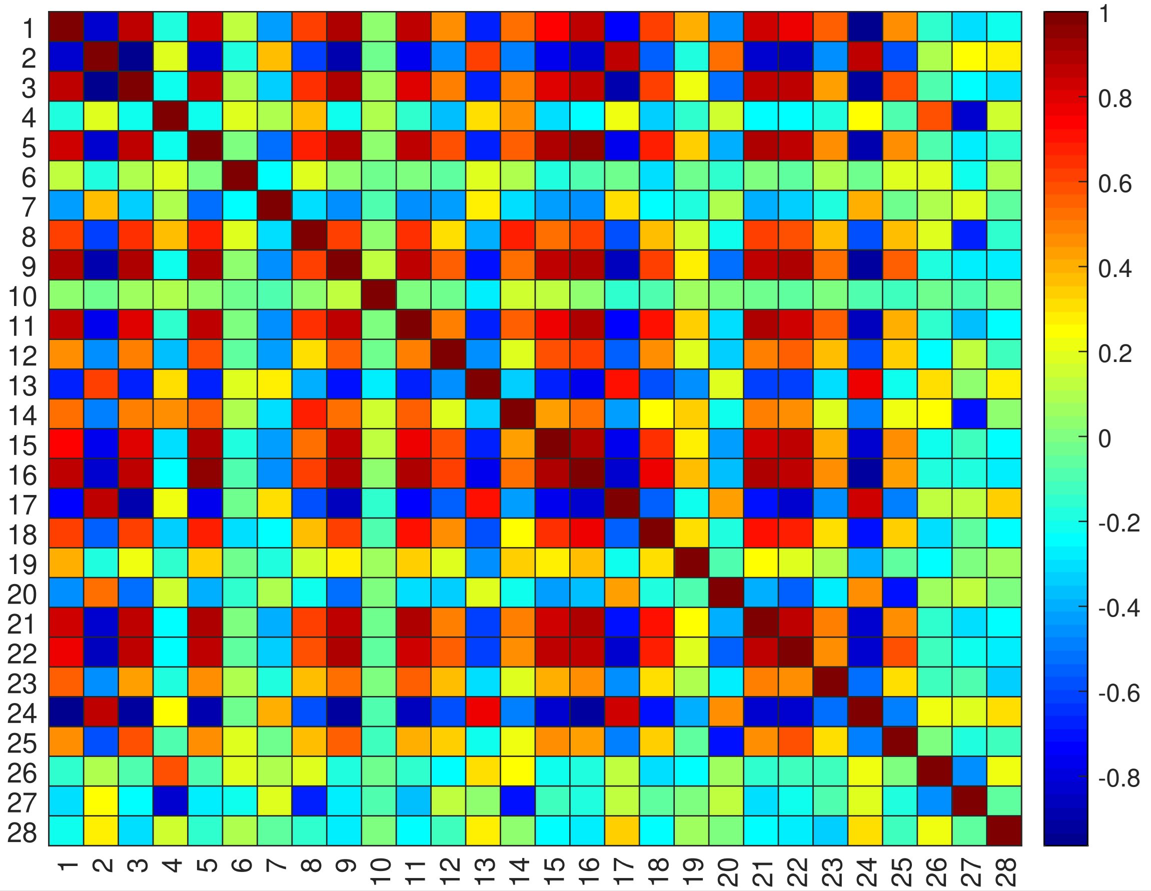

Variable labels

1.	Life expectancy	6.	Population density	11.	Health coverage	16.	Gov.h.spending	21.	Digitalization	26.	Comorbidities
2.	Young population	7.	Rural population	12.	Migration	17.	Household size	22.	Medical resources	27.	Soc. Connectedness
3.	Elderly population	8.	Int. tourism	13.	Air pollution	18.	Overweight prevalence	23.	Immunization	28.	Gender
4.	Population	9.	Education	14.	Air transport	19.	Asthma prevalence	24.	Mortality		
5.	Econ. development	10.	Labor market	15.	Sus. development	20.	Income inequality	25.	Weather		

Figure S1. Correlation matrix.

S2.1 Individual indices

Medical resources index: The Medical resources index is estimated as a Principal Component Analysis (PCA) weighted index of the logs of three variables[2]. These are:

- Physicians (per 1,000 people)

- Nurses and midwives (per 1,000 people).

- Hospital beds (per 1,000 people).

Non-natural causes mortality index: The Non-natural causes mortality index is calculated as a Principal Component Analysis (PCA) weighted index of the logs of these four variables found in WDI:

- Mortality rate attributed to household and ambient air pollution, age-standardized (per 100,000 population).

- Cause of death, by communicable diseases and maternal, prenatal and nutrition conditions (% of total).

- Mortality from CVD, cancer, diabetes or CRD between exact ages 30 and 70, female (%).

- Mortality rate attributed to unsafe water, unsafe sanitation and lack of hygiene (per 100,000 population).

Immunization index: The Immunization index is estimated as a Principal Component Analysis (PCA) weighted index of the logs of two variables:

- Immunization, DPT (% of children ages 12-23 months).
- Immunization, measles (% of children ages 12-23 months).

Comorbidities index: The Comorbidities index is calculated as a Principal Component Analysis (PCA) weighted index of the thirtheen individual measures describing the burdens of disease, measured by a metric called 'Disability Adjusted Life Years' (DALYs). These are:

- Neglected tropical diseases and malaria.
- Maternal disorders.
- Neonatal disorders.
- Nutritional deficiencies.
- Neoplasms.
- Cardiovascular diseases.
- Chronic respiratory diseases.
- Cirrhosis and other chronic liver diseases.
- Digestive diseases.
- Neurological disorders.
- Mental and substance use disorders.
- Musculoskeletal disorders.
- Other non-communicable diseases.

Social connectedness index: The original social connectedness index (SCI) was introduced in[3] as a measure of the magnitude of Facebook connections between pairs of countries i and j. Formally, the ij-th index is estimated as

$$\text{Social Connectedness}_{ij} = \frac{\text{FB Connections}_{ij}}{\text{FB Users}_i \times \text{FB Users}_j}, \tag{S3}$$

where FB Connections$_{ij}$ is the total number of Facebook connections between i and j and FB Users$_l$ is the number of Facebook users in country l. Combining all pairs, this results in an $N \times N$ dimensional matrix. We transform it to be an only one-country measure by estimating the log of the PageRank (eigenvector centrality) of each country in the original SCI matrix[4].

Digitalization index: The Immunization index is estimated as a Principal Component Analysis (PCA) weighted index of the logs of four variables:

- Individuals using the Internet (% of population).
- Fixed broadband subscriptions (per 100 people).
- Fixed telephone subscriptions (per 100 people).
- Mobile cellular subscriptions (per 100 people).

S3 Bayesian model averaging

We specify our linear regression model M_m for the final number of COVID-19 infections/death rates of the first wave as

$$y_i = \beta_0 + \beta_m^T \mathbf{X}_i^m + \gamma s_i + \delta d_i + u_i, \tag{S4}$$

where, for simplicity, we denote both the log of registered COVID-19 infections per million population and the log of COVID-19 deaths per million population of country i as y_i. In the equation, $\mathbf{X}_i^m$ it is a k_m dimensional vector of health, social and economic explanatory variables that determine the dependent variable, β_m is the vector describing their marginal contributions, β_0 is the intercept of the regression, and u_i is the error term. The s_i term controls for the impact of government responses, and γ is its coefficient. Finally, we also include the term d_i, with δ capturing its marginal effect, that measures the duration of the pandemics within the economy. This allows us to control for the possibility that the countries are in a different state of the disease spreading process.

BMA leverages Bayesian statistics to account for model uncertainty by estimating each possible model M_m, and thus evaluating the posterior distribution of each parameter value and probability that a particular model is the correct one[5]. More precisely, in BMA, the posterior probability for the parameters $g(\beta_m|y,M_m)$ is calculated using M_m as:

$$g(\beta_m|y,M_m) = \frac{f(y|\beta_m,M_m)g(\beta_m|M_m)}{f(y|M_m)}. \tag{S5}$$

It is clear that the posterior probability is proportional to $f(y|\beta_m,M_m)$, - the likelihood of seeing the data under model M_m with parameters β_m, and $g(\beta_m|M_m)$ – the prior distribution of the parameters included in the proposed model. By assuming a prior model probability $P(M_m)$, we can implement the same rule to evaluate the posterior probability that model M_m is the true one, as

$$P(M_m|y) = \frac{f(y|M_m)P(M_m)}{f(y)} = \frac{f(y|M_m)P(M_m)}{\sum_{n=1}^{2^k} f(y|M_n)P(M_n)}. \tag{S6}$$

The term $f(y|M_m)$ is called the marginal likelihood of the model and is used to compare different models to each other. The posterior model probability can also be written as

$$P(M_m|y) = \frac{B_{m0}P(M_m)}{\sum_{n=1}^{2^k} B_{n0}P(M_n)}, \tag{S7}$$

where B_{m0} is the Bayes information criterion between model M_m and the baseline model M_0. In our case this is the model including government social distancing measures and the length of the coronavirus crisis in the country.

With this setup, we can define the posterior distribution of β as a weighted average of the posterior distributions of the parameters under each model using the posterior model probabilities as weights

$$g(\beta|y) = \sum_{j=1}^{2^k} g(\beta|y,M_m)P(M_m|y). \tag{S8}$$

Here, we are interested only in some parameters of the posterior distribution, such as the posterior mean and variance of each parameter. Using equation (S8) we can calculate the posterior mean as:

$$\mathbb{E}\left[(\beta|y\right] = \sum_{m=1}^{2^k} \mathbb{E}\left[(\beta|y,M_m\right] P(M_m|y), \tag{S9}$$

and the posterior variance as:

$$\mathrm{var}\left[(\beta|y\right] = \sum_{m=1}^{2^k} \mathrm{var}\left[(\beta|y,M_m\right] P(M_m|y) + \sum_{m=1}^{2^k} P(M_m|y)\left(\mathbb{E}\left[(\beta|y,M_m\right] - \mathbb{E}\left[(\beta|y,\right]\right)^2. \tag{S10}$$

Since the posterior mean is a point estimate of the average marginal contribution, we use it as our measure of the effect of the correlate on the COVID-19 impact.

Another interesting statistic is the posterior inclusion probability PIP_h of a variable h, which measures the posterior probability that the variable is included in the 'true' model. Mathematically, PIP_h is defined as the sum of the posterior model probabilities for all of the models that include the variable:

$$PIP_h = (P(\beta_h \neq 0) = \sum_{m:\beta_h\neq 0}^{2^k} P(M_m|y). \tag{S11}$$

Posterior inclusion probabilities offer a more robust way of determining the effect of a variable in a model, as opposed to using p-values for determining statistical significance of a model coefficient because they incorporate the uncertainty of model selection.

According to equations (S5) and (S6), it is clear that we need to specify priors for the parameters of each model and for the model probability itself. To keep the model simple and easily implemented here we use the most often implemented priors. In other words, for the parameter space we elicit a prior on the error variance that is proportional to its inverse, $p(\sigma^2) \approx 1/\sigma^2$, and a uniform distribution on the intercept, $p(\alpha) \to 1$, while the Zellner's g-prior is used for the β_m parameters, and for the model space we utilise the Beta-Binomial prior. To estimate the posterior parameters we use a Markov Chain Monte Carlo (MCMC) sampler, and report results from a run with 200 million recorded drawings and after a burn-in of 100 million discarded drawings. Finally, before we perform the inference the data for each variable is transformed into its z-score, in order to normalize the measuring unit. The theoretical background behind our setup can be read in Refs.[5–8].

S4 Robustness checks

S4.1 BMA outliers check

As said in the main text, we check the robustness of our results against the presence of outliers by removing a country from the sample and re-performing the BMA procedure with the resulting countries. We repeat this procedure for every country and recover the median results for

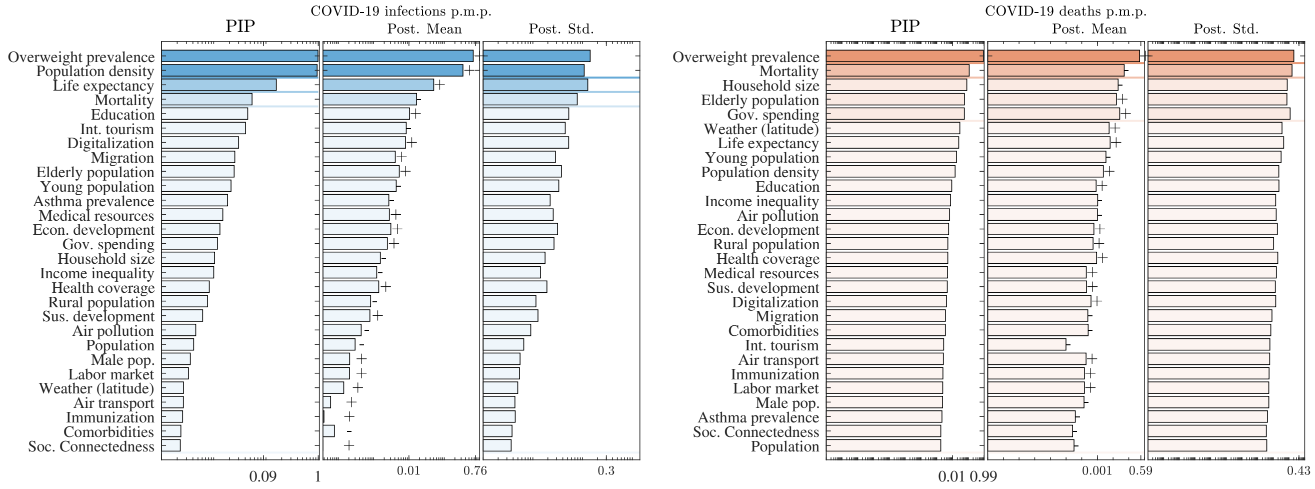

Figure S2. BMA outliers check. Bars for the posterior inclusion probability (PIP), posterior mean (Post. Mean) and the posterior standard deviation (Post. Std.) of each potential correlate. The variables are ordered according to their PIP. The Post. Mean is in absolute value. The signs next to the bar of each variable indicate the direction of its impact. The horizontal lines divide the variables into groups according to their PIP. The horizontal axis is on a logarithmic scale. The setup for the estimation is described in SI Section S3.

each potential correlate. The results can be seen in Fig. S2. They are nearly identical to the ones presented in the main text, thus suggesting that our results are robust to outliers.

Table S4 outlines the countries which had the biggest impact on the observed credibility of a given correlate. We define two types of countries, i) the weakest contributor, this is the country which when excluded from the sample leads to the largest PIP for the studied correlate; and ii) the strongest contributor – i.e., the country which when excluded we observe the lowest PIP for the studied correlate. We find numerous countries which can be significant contributors for each correlate, thus indicating that there is indeed heterogeneity in the socio-economic features of the countries.

Variable	COVID-19 infections p.m.p. Weakest contributor	COVID-19 infections p.m.p. Strongest contributor	COVID-19 deaths p.m.p. Weakest contributor	COVID-19 deaths p.m.p. Strongest contributor
**Healthcare Infrastructure**				
Medical resources	Trinidad & Tobago	Bolivia	Peru	Ireland
Health coverage	Peru	Sweden	Peru	Sweden
**National health statistics**				
Life expectancy	Namibia	USA	Peru	Sweden
Mortality	Peru	Jamaica	Peru	Jamaica
Immunization	Jamaica	Peru	Sweden	Norway
Overweight prevalence	USA	Peru	Jamaica	Peru
Asthma prevalence	Peru	Bolivia	Sweden	Peru
**Economic performance**				
Economic development	Rwanda	Bolivia	Peru	Russia
Labor market	USA	Trinidad & Tobago	Russia	Mozambique
Government spending	Rwanda	Jamaica	Peru	Jamaica
Income inequality	USA	Brazil	Jamaica	Brazil
**Societal characteristics**				
Social connectedness	USA	Italy	Sweden	Peru
Digitalization	Peru	USA	Jamaica	Mozambique
Education	Rwanda	Ireland	Peru	Australia
Household size	Italy	Ireland	Peru	Ireland
**Demographic structure**				
Elderly population	Italy	Rwanda	Italy	Sweden
Young population	Italy	Sweden	Italy	Sweden
Gender	USA	Peru	Mauritius	Slovakia
Rural population	Rwanda	Italy	Russia	Sweden
Migration	USA	Pakistan	Italy	Jamaica
Population density	USA	Italy	Australia	Russia
**Natural environment**				
Sustainable development	USA	Togo	Italy	Mauritius
Air Pollution	USA	Peru	Indonesia	Peru
Air transport	Jamaica	Peru	Sweden	Mozambique
International Tourism	Moldova	Peru	Bolivia	Ethiopia
Weather (latitude)	Jamaica	Rwanda	Australia	Chile

Table S4. **Contributors to the credibility of a correlate.**

S4.2 Alternate end date of the first wave

In this robustness check, we change the end date of the pandemic to be equal to the first date after the day at which the daily government response index is at its maximum and that is at least 20% lower than the daily maximum. This effectively prolongs the duration of the first wave. The results are shown in Fig. S3. In this case, it appears that there are more variables that are either strong or medium correlates of the COVID-19 infections/death rates. Nonetheless, the variables which were found in the main results, persist in being correlates with strong or medium evidence.

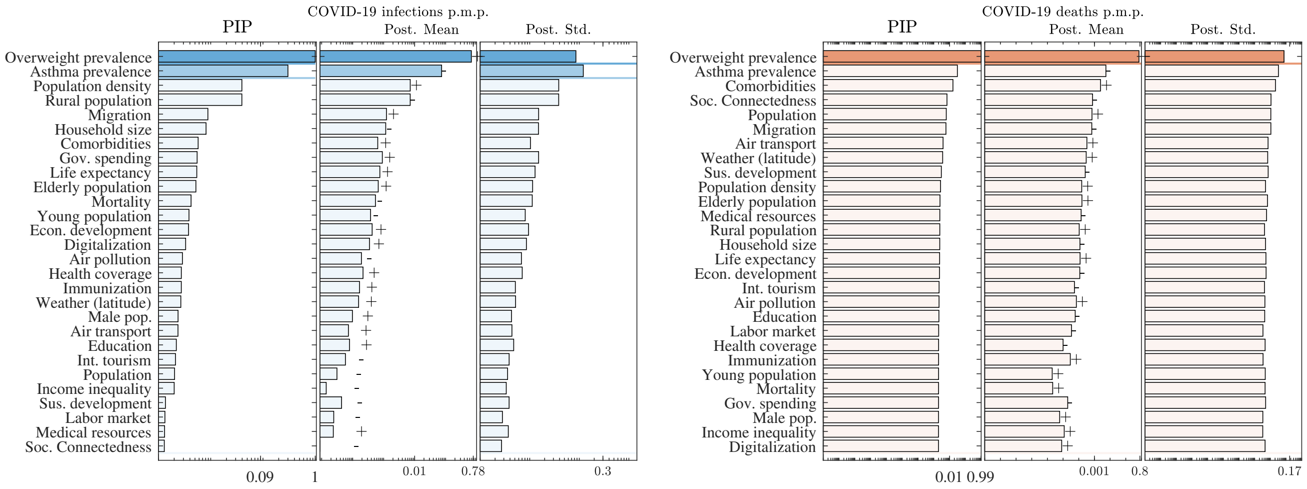

Figure S3. BMA robustness results. The end date is the first time the gov. response index is 20% lower than its maximum. Bars for the posterior inclusion probability (PIP), posterior mean (Post. Mean) and the posterior standard deviation (Post. Std.) of each potential correlate. The variables are ordered according to their PIP. The Post. Mean is in absolute value. The signs next to the bar of each variable indicate the direction of its impact. The horizontal lines divide the variables into groups according to their PIP. The horizontal axis is on a logarithmic scale. The setup for the estimation is described in SI Section S3.

S4.3 BMA quasi-Poisson specification

In this check, we change the dependent variable to be the raw number of infections and deaths at the end of the first wave. That is, now the dependent variable describes counts and the linear regression framework is not a suitable model. Instead, for the estimation of the marginal impact we use a quasi-Poisson model. This is the most often used procedure when the dependent variable is given as a count that has a large variance[9]. Indeed, the number of COVID-19 infections or deaths has a variance larger than its mean. This is apparently due to the disparate effect the pandemic had throughout the world.

The results can be seen in Fig. S4. Again, the variables that were found to be strong correlates with the COVID-19 infections and mortality rates, remain strong correlates even in this specification. Thus, it can be concluded that our results are robust to a different model specification.

S4.4 BMA Spatial autocorrelation check

In the last check we add a spatial weighting matrix in the baseline model in order to account for the potential spatial autocorrelation (SAR) in the spread of COVID-19. Multiple studies have indicated that this effect might exist (See for example[10]). Again our findings do not significantly change.

The SAR model which we use, takes the following matrix form

$$\mathbf{y} = \rho \mathbf{W}\mathbf{y} + \gamma \mathbf{s} + \mathbf{d}\delta + \varepsilon, \tag{S12}$$

Where $\mathbf{y}$ is the n-dimensional vector of infection or mortality rates, $\mathbf{s}$ and $\mathbf{d}$ are matrices containing the baseline independent variables and $\mathbf{W}$ is a known row-standardized spatial weight distance matrix between the studied countries. The parameter ρ is a coefficient on the spatially lagged dependent variable, $\mathbf{Wy}$. The spatial weight matrix, $\mathbf{W}$, is $n \times n$ stochastic matrix, where n is the number of countries with element w_{ij} defining the spatial relations between locations i and j. This matrix is constructed through the following steps:

1. Gather data for the latitude and longitude of each county from Google Developers.
2. Calculate the Haversine distance D_{ij} between each pair of countries i and j using the data. This procedure allows us to determine the great-circle distance between any two countries on a sphere given their longitudes and latitudes (See Ref.[11]).
3. Construct a distance matrix, $\mathbf{D} = [D_{ij}]$, between the countries using the estimations from the previous step.

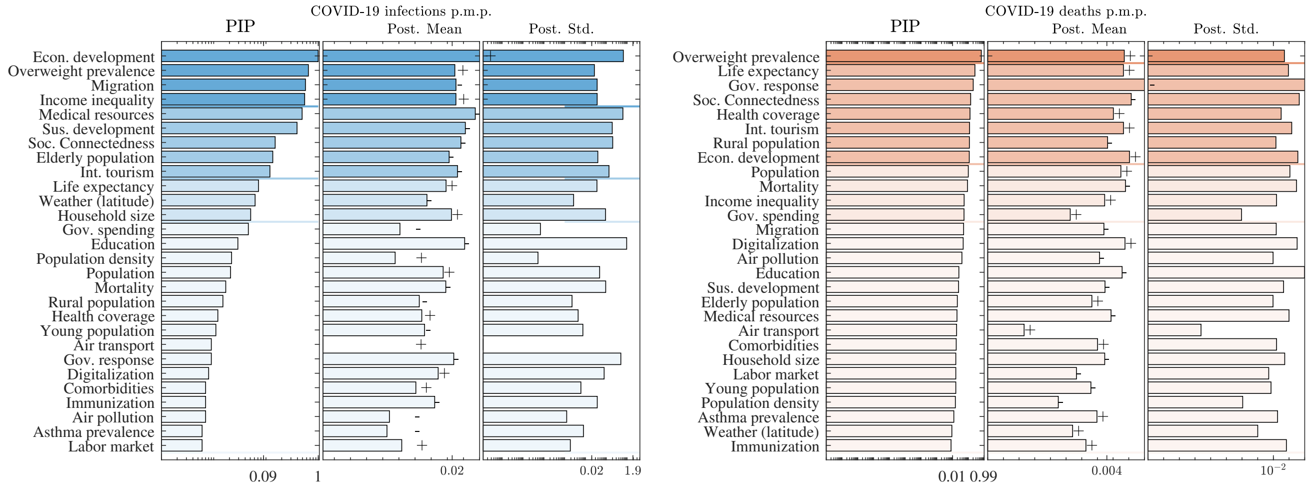

Figure S4. BMA robustness results. The link function used in the model is quasi-Poisson. Bars for the posterior inclusion probability (PIP), posterior mean (Post. Mean) and the posterior standard deviation (Post. Std.) of each potential correlate. The variables are ordered according to their PIP. The Post. Mean is in absolute value. The signs next to the bar of each variable indicate the direction of its impact. The horizontal lines divide the variables into groups according to their PIP. The horizontal axis is on a logarithmic scale. The setup used to estimate the results is described in SI Section S3.

4. Row-standardize the distance matrix to obtain the spatial weight matrix, $\mathbf{W}$. That is, the ij-th entry of the $\mathbf{W} = [W_{ij}]$ is $W_{ij} = D_{ij}/sum_j D_{ij}$.

The baseline SAR results are presented in Table S5. We observe that the spatial autocorrelation coefficient estimate for the SAR model is negative and statistically significant when the dependent variable is the infection rate, indicating the presence of spatial autocorrelation in the regression relationship. The coefficient remains negative when the dependent variable is the mortality rate, though it loses its significance.

	**COVID 19 infections p.m.p.**		**COVID 19 deaths p.m.p.**	
**Variable**	**Coefficient**	**p-value**	**Coefficient**	**p-value**
Gov. response (log)	-3.09	0.00∗	-1.46	0.00*
Days since first local case	0.02	0.00∗	0.02	0.00*
Days since first global case	0.05	0.00∗	0.01	0.51
Distance-based spatial weights	-0.81	0.03∗	-1.00	0.13

Table S5. SAR results. $*$ indicates significance at $\alpha = 0.05$

The results for the BMA after implementing SAR as a baseline model, can be seen in Fig. S5. They are nearly identical to the ones presented in the main text, thus suggesting that our results are robust after accounting for spatial autocorrelation.

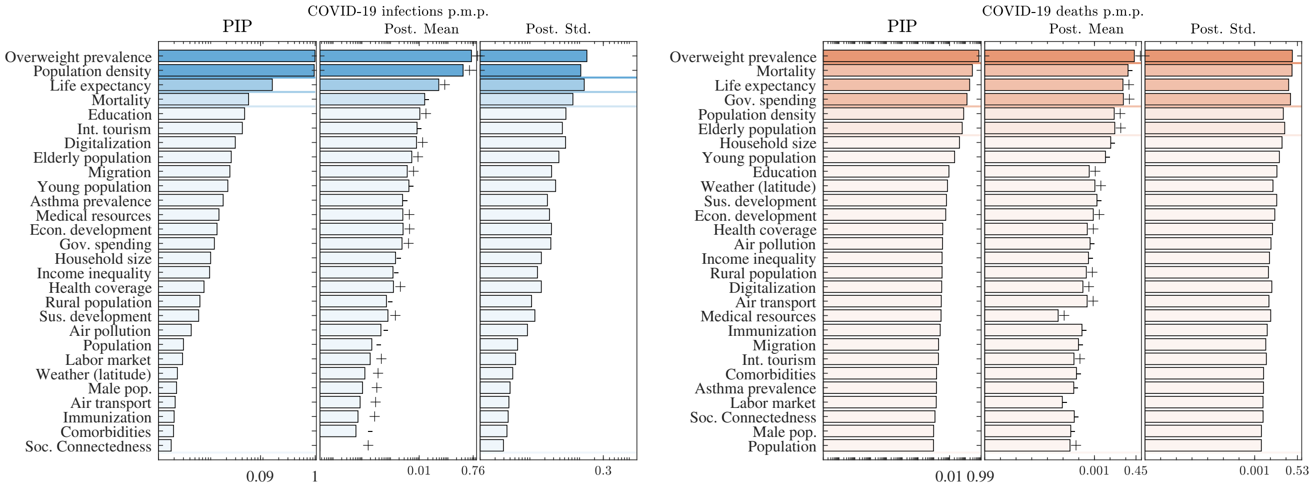

Figure S5. **BMA robustness results. The base model includes Spatial Autoregressive coefficient.** Bars for the posterior inclusion probability (PIP), posterior mean (Post. Mean) and the posterior standard deviation (Post. Std.) of each potential correlate. The variables are ordered according to their PIP. The Post. Mean is in absolute value. The signs next to the bar of each variable indicate the direction of its impact. The horizontal lines divide the variables into groups according to their PIP. The horizontal axis is on a logarithmic scale. The setup used to estimate the results is described in SI Section S3.

S5 Construction of the coronavirus correlates Jointness space

To construct the coronavirus correlates Jointness space we utilize a network approach. In this network, the nodes represent the potential health, social and economic correlates, whereas the edge between a pair of correlates is given by a Jointness measure of the posterior probability that the pair is included in the same model explaining the COVID-19 infections/mortality rates. As a Jointness measure we utilize the the Hofmarcher et al. Jointness test. This test is a regularised version of the well-known Yule's Q association coefficient and is derived based on an augmented contingency table of variable inclusion. The table allows us to avoid the problems that arise due to zero counts[12]. The test statistic, J_{hk} between variables h and k, is calculated as

$$J_{hk} = \frac{(a+\frac{1}{2})(d+\frac{1}{2}) - (b+\frac{1}{2})(c+\frac{1}{2})}{(a+\frac{1}{2})(d+\frac{1}{2}) + (b+\frac{1}{2})(c+\frac{1}{2})}, \tag{S13}$$

where a,b,c and d are the empirical counts of the MCMC drawings in which, respectively, h and k are included together; h is included and k is excluded; h is not included and k is included; and both h and k are excluded. The main advantage of this test over other jointness measures is that it is appropriately defined as long as one of the studied variables is included in the true model with positive probability. Moreover, it is monotonic, with larger values implying that the two variables are complements; commutative,i.e. $J_{hk} = J_{kh}$; it is bounded between -1, and 1, and has an adequate limiting behavior.

To visualize the resulting network we use only the positive links (those that are greater than 0). To set the coordinates of each node we use the Force-Layout drawing algorithm.

References

1. Hale, T., Petherick, A., Phillips, T. & Webster, S. Variation in government responses to covid-19. *Blavatnik school government working paper* **31** (2020).
2. Vyas, S. & Kumaranayake, L. Constructing socio-economic status indices: how to use principal components analysis. *Heal. policy planning* **21**, 459–468 (2006).
3. Bailey, M., Cao, R., Kuchler, T., Stroebel, J. & Wong, A. Social connectedness: Measurement, determinants, and effects. *J. Econ. Perspectives* **32**, 259–80 (2018).
4. Bonacich, P. Some unique properties of eigenvector centrality. *Soc. networks* **29**, 555–564 (2007).
5. Moral-Benito, E. Model averaging in economics: An overview. *J. Econ. Surv.* **29**, 46–75 (2015).
6. Fernandez, C., Ley, E. & Steel, M. F. Model uncertainty in cross-country growth regressions. *J. applied Econom.* **16**, 563–576 (2001).
7. Fernandez, C., Ley, E. & Steel, M. F. Benchmark priors for bayesian model averaging. *J. Econom.* **100**, 381–427 (2001).
8. Ley, E. & Steel, M. F. On the effect of prior assumptions in bayesian model averaging with applications to growth regression. *J. applied econometrics* **24**, 651–674 (2009).

9. Ver Hoef, J. M. & Boveng, P. L. Quasi-poisson vs. negative binomial regression: how should we model overdispersed count data? *Ecology* **88**, 2766–2772 (2007).

10. Krisztin, T., Piribauer, P. & Wögerer, M. The spatial econometrics of the coronavirus pandemic. *Lett. Spatial Resour. Sci.* **13**, 209–218 (2020).

11. Robusto, C. C. The cosine-haversine formula. *The Am. Math. Mon.* **64**, 38–40 (1957).

12. Hofmarcher, P., Cuaresma, J. C., Grun, B., Humer, S. & Moser, M. Bivariate jointness measures in bayesian model averaging: solving the conundrum. *J. Macroecon.* **57**, 150–165 (2018).

Variable	Measure	Mean	Std.
Coronavirus outcome	Coronavirus infections p.m.p.	5.92	2.20
	Coronavirus deaths p.m.p.	2.58	2.20
Government response	Government response index	−1.84	0.37
Epidemic duration	Days since first registered local case[a]	99.97	61.43
	Days since first global case[a]	58.22	19.20
**Healthcare Infrastructure**			
Medical resources	Medical resources index[b]	0.10	1.06
Health coverage	UHC service coverage index	4.18	0.24
**National health statistics**			
Life expectancy	Life expectancy at birth, (years)	4.29	0.10
Mortality	Non-natural causes mortality index[b]	−0.30	1.04
Immunization	Immunization index[b]	0.15	0.66
Comorbidities	Comorbidities index[b]	0.35	3.68
Overweight prevalence	% of adults with BMI $> 25\ kg/m^2$	3.81	0.38
Asthma prevalence	Asthma prevalence (% of population)	1.53	0.33
**Economic performance**			
Economic development	GDP p.c., PPP $	9.58	1.08
Labor market	Employment to population ratio (%)	4.02	0.20
Government spending	Gov. health spending p.c., PPP $[c]	5.85	1.71
Income inequality	GINI index	3.61	0.21
**Societal characteristics**			
Social connectedness	Social connectedness index (PageRank)[b]	−0.79	1.31
Digitalization	Digitalization index[b]	0.11	0.95
Education	Human capital index	−0.55	0.27
Household size	Avg. no. of persons in a household	1.25	0.32
**Demographic structure**			
Elderly population	Population age 65+ (% of total)	2.12	0.74
Young population	Population ages 0-14 (% of total)	3.17	0.40
Gender	50%+ male population	/	/
Rural population	Rural population (% of total)	3.38	1.21
Migration	Int. migrant stock (% of population)	1.09	1.47
Population density	People per sq. km	4.34	1.32
Population size	Population, total	16.69	1.41
**Natural environment**			
Sustainable development	Ecological Footprint (gha/person)	0.98	0.65
Air Pollution	Yearly avg P.M. 2.5	3.01	0.63
Air transport	Yearly passengers carried	8.22	2.74
International Tourism	Number of tourist arrivals	15.20	1.54
Weather (latitude)	Geographic coordinate: Latitude	21.65	26.85

Table S3. Summary statistics.
[a] Raw values.
[b] Individual calculations.
[c] 10 year averages.
